# Surgical treatment as a key determinant of outcome in phosphaturic mesenchymal tumors of the bone and soft tissue: a systematic review and case series

**DOI:** 10.1530/EOR-2025-0100

**Published:** 2025-11-03

**Authors:** Julian P Maier, Moritz A Krapohl, Georg W Herget, Kilian Reising, Hannah Füllgraf, Peter Bronsert, David Braig, Hagen Schmal

**Affiliations:** ^1^ Department of Orthopedics and Trauma Surgery, University Medical Center Freiburg, Freiburg, Germany; ^2^ Berta-Ottenstein-Program, Faculty of Medicine, University of Freiburg, Freiburg, Germany; ^3^ Faculty of Medicine, University of Freiburg, Freiburg, Germany; ^4^ Comprehensive Cancer Center Freiburg (CCCF), University Medical Center Freiburg, Freiburg, Germany; ^5^ Department of Pathology, University Medical Center Freiburg, Freiburg, Germany; ^6^ Department of Plastic and Hand Surgery, University Medical Center Freiburg, Freiburg, Germany; ^7^ Department of Orthopedic Surgery, University Hospital Odense, Odense, Denmark

**Keywords:** phosphaturic mesenchymal tumor, bone tumors, soft tissue tumors, FGF-23, surgical resection, tumor-induced osteomalacia

## Abstract

**Purpose:**

- Phosphaturic mesenchymal tumors (PMTs) are rare neoplasms causing tumor-induced osteomalacia (TIO), usually through fibroblast growth factor 23 (FGF-23) secretion. They arise in bone or soft tissue and present with non-specific symptoms, often leading to delayed diagnosis. This study evaluates tumor characteristics, the role of surgical therapy, and its impact on outcomes while highlighting key aspects of PMT management.

**Methods:**

- A systematic review was conducted according to PRISMA guidelines, including 188 studies and 584 PMT cases, focusing on clinical presentation, treatment modalities, and outcomes. In addition, we retrospectively analyzed four patients treated at our institution.

**Results:**

- The mean patient age was 49 ± 15 years, with a slight male predominance (56%). Tumors were evenly distributed between bone and soft tissue, most commonly affecting the lower extremities. Frequent symptoms included pain (90%) and pathological fractures (69%). Elevated FGF-23 levels were detected preoperatively in over 90% of cases. Complete remission was achieved in 66.2% of surgically treated patients compared to 29.4% of non-operatively managed patients (*P* < 0.001). Complete resection was associated with significantly higher remission rates (55.7 vs 6.6%, *P* = 0.001), particularly in bone-localized PMT. In our case series, all patients undergoing complete resection showed no evidence of disease and relief of TIO-associated symptoms.

**Conclusions:**

- PMTs often lead to diagnostic delays due to non-specific symptoms. Complete surgical resection is the strongest predictor of favorable outcomes, especially for bone-localized tumors. An interdisciplinary approach is critical for early diagnosis, optimal treatment, and maximizing favorable clinical outcomes.

## Introduction

Phosphaturic mesenchymal tumors (PMTs) are rare, predominantly benign neoplasms commonly associated with tumor-induced osteomalacia (TIO). This paraneoplastic syndrome is primarily driven by fibroblast growth factor 23 (FGF-23) secretion, leading to renal phosphate wasting and impaired bone mineralization. Among various neoplasms that can potentially cause TIO, PMTs are the most frequently implicated (1). Their excessive FGF-23 production directly inhibits phosphate reabsorption in the renal proximal tubules and reduces the synthesis of 1,25-dihydroxyvitamin D (2). PMTs typically arise in bone or soft tissue and often present with non-specific symptoms such as bone pain, reduced mobility, and pathological fractures, which can occur at virtually any site and are not necessarily indicative of the tumor’s location (3, 4). An exception in the localization of PMTs is parenchymal or intra- and retroperitoneal organs, where none of these tumors – except for metastasis – have primarily been reported (1, 5, 6, 7, 8, 9, 10). Most tumors occur in middle-aged adults, whereas a smaller number have been reported in infants or the elderly (11).

Despite increasing recognition, the number of reported PMT cases in the literature remains low, primarily as case reports or smaller case series (11). Furthermore, most available studies focus on these tumors’ histopathologic, immunohistochemical, or radiological features (6). Despite recent advances in diagnostic and therapeutic modalities, the management of PMTs remains challenging, with an average diagnostic delay of several years (12, 13, 14, 15, 16). In addition, evidence regarding the optimal surgical treatment of PMTs is limited, and comprehensive reports on surgical outcomes are lacking, highlighting the need for clear clinical recommendations.

This study aims to assess the clinical characteristics and challenges of managing PMTs. Through a systematic review, we analyzed one of the largest cohorts of PMTs reported in the literature, specifically evaluating the role of surgical therapy and its impact on clinical outcomes. In addition, we present a case series of PMT patients treated at our institution, providing supplementary insights into clinical management and treatment responses.

## Methods

Ethical approval was obtained from the local ethics committee of the University of Freiburg before the study commenced (protocol 25-1526-S1-retro). Written informed consent was obtained from all patients, and all methods were conducted following the approved guidelines. Clinical data from four patients diagnosed with PMT, treated at the the Department of Orthopedics and Trauma Surgery and Department of Plastic and Hand Surgery, University Medical Center Freiburg, Germany, between 2020 and 2023, were included in the case series.

### Search strategy and inclusion criteria

**Figure 1:**
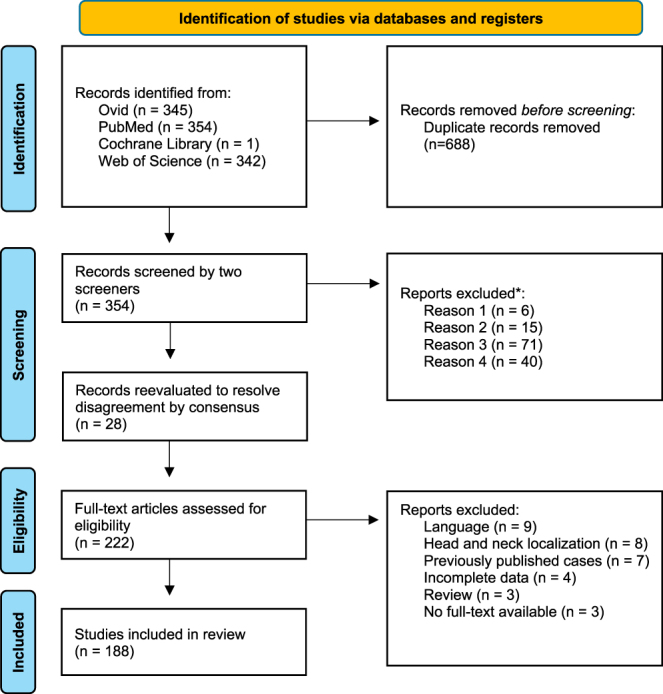
PRISMA flow diagram of study selection. Out of 1,042 identified records, 688 duplicates were removed. After screening and full-text assessment, 188 studies met the inclusion criteria and were included in the review. Reasons for exclusion are detailed at each stage. Figure visualized according to (17).

A systematic review was conducted in accordance with the Preferred Reporting Items for Systematic Reviews and Meta-Analyses (PRISMA) guidelines (17) and submitted to PROSPERO (ID: CRD42024516997), a database for registered systematic reviews and meta-analyses. Relevant studies were extracted from medline’s electronic databases (via PubMed, Ovid, and Web of Science) and the Cochrane Library. The systematic search strategy is shown in Fig. 1, with the final search term: (phosphaturic*) AND ((mesenchym*) OR (soft tissue) OR (oss*) OR (bone)) AND ((neoplasm*) OR (tumor*) OR (tumour*)) AND ((osteomalacia*) OR (ricket*)). Data collection was performed between 01/09/2022 and 22/04/2023.

Studies were included if they were published in English or German and fulfilled the criteria as case reports, case series, or patient cohorts with a diagnosed PMT. Exclusion criteria included studies published before 1985, previous systematic reviews (to avoid duplication), publications unrelated to the topic, incorrect diagnoses, and cases in which tumors were not localized or further specified. PMTs of the head and neck region were excluded due to their distinct biological behavior and specific clinical management, as previously described by others (18, 19).

In addition, we retrospectively identified patients with a histopathological diagnosis of PMT from our institutional database. Inclusion criteria required a diagnosis confirmed by a board-certified pathologist and a minimum follow-up of 6 months. A total of four patients met these criteria and were included in the presented case series.

### Data extraction

A total of 1,042 studies were identified, with 688 records removed before screening due to duplication. Subsequent screening was independently conducted by two authors (JPM and MAK). During this process, 132 studies were excluded based on the selection criteria, while 28 studies required reevaluation to resolve disagreements between the screeners by consensus. After screening, a full-text assessment was performed on 222 articles to determine final eligibility. Following the exclusion of an additional 34 studies based on selection criteria, 188 studies comprising a total of 584 patients were included in the review (Fig. 1). The authors recorded publication details, including author names, year of publication, and the number of patients. Extracted data included patient demographics, clinical presentation, biomarkers, imaging diagnostics, treatment procedures, and clinical outcomes – defined as symptom remission. Notably, data were not available for every parameter in all patients. Thus, patients with missing data on specific parameters were excluded from further sub-analysis, and results should be interpreted accordingly. The number of patients with available data for individual parameters is stated as ‘*n*’ in the results section and can vary for different analyses.

Patients included in the case series were manually reviewed by two independent authors to collect demographic data, laboratory and imaging findings, treatment details, and patient outcomes. Collected demographic and clinical parameters included age at diagnosis, sex, tumor location, clinical symptoms, and diagnostic data. Treatment and outcome parameters included treatment type, resection margins, pathological reports, and follow-up data. Full remission was defined as complete resolution of clinical symptoms following treatment. Partial remission was defined as improvement of clinical symptoms without full normalization.

Laboratory reference ranges at our institution were as follows: serum phosphorus (0.81–1.45 mmol/L), serum calcium (2.20–2.55 mmol/L), alkaline phosphatase (35–105 U/L), and serum FGF-23 (25–110 kRU/L).

### Risk of bias assessment

Since most included studies were case reports and case series, assessing the risk of bias would be challenging. Given the descriptive nature of our study and the rarity of PMTs, a formal risk of bias assessment may not have accurately reflected potential biases and was therefore not performed. The presented data should be interpreted accordingly.

### Statistical analysis

For data collection, synthesis, and analysis, Microsoft Excel (Version 16.94, Microsoft Corp., USA) and IBM SPSS (Version 30.0.0.0, USA) were used. The recorded items relevant to the investigated subject were categorized in an Excel spreadsheet for further analysis. The Shapiro–Wilk test was performed to assess normality and homogeneity of the dataset. If normality was not met, Mann–Whitney U testing was used for post-hoc analyses. Pearson chi-square test was applied to evaluate significant associations between two categorical variables. Cramér’s V was calculated following significant chi-square testing to quantify the strength of association between nominal categorical variables.

## Results

### Epidemiology, tumor localization, and clinical characteristics

**Figure 2:**
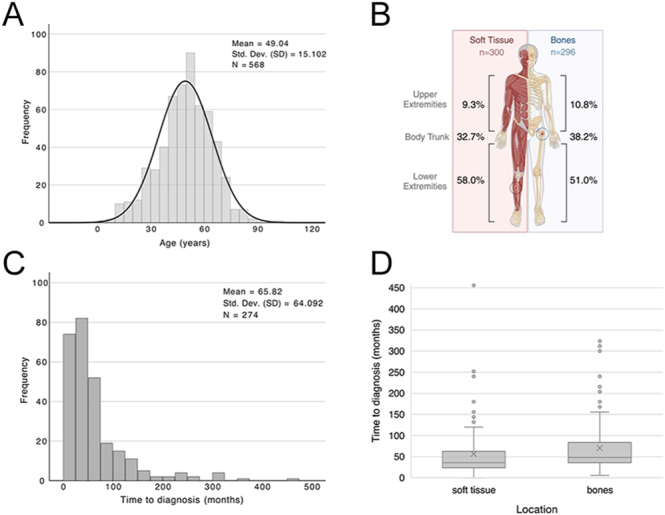
Patient characteristics and diagnostic delay in soft tissue and bone tumors. (A) Age distribution of patients (*n* = 568) with a mean age of 49.04 years (SD = 15.10). (B) Anatomical distribution of tumor location in soft tissue (*n* = 300) and bone (*n* = 296) cases. Most soft tissue or bone-localized PMT tumors occurred in the lower extremities. (C) Time to diagnosis for a subset of patients (*n* = 274) with a mean delay of 65.82 months (SD = 64.09). (D) Comparison of time to diagnosis in PMTs of the soft tissue (*n* = 124) and bones (*n* = 125). Bone-localized PMT tumors show a significantly longer diagnostic delay compared to soft tissue tumors (*P* = 0.012). Boxplot showing median, interquartile range, and outliers. Parts of this figure (B) were created in BioRender, Maier J (2025) (https://BioRender.com/uwy1qk3).

Among the 584 PMT cases analyzed, the mean age at diagnosis was 49 ± 15 years (*n* = 568), with a slight male predominance (56% male vs 44% female, *n* = 574), Fig. 2A. Pain (90.2%, *n* = 277, *n* = 307) and pathological fractures (69%, *n* = 202, *n* = 290) were the most prevalent clinical symptoms, followed by further non-specific symptoms such as muscle weakness (53%, *n* = 151, *n* = 283) and restricted mobility (44%, *n* = 119, *n* = 272). Other rare symptoms are listed in Supplementary Table 1A (see section on Supplementary materials given at the end of the article).

PMTs were evenly distributed between soft tissue (*n* = 300) and bone (*n* = 296), with the lower extremities being the most affected region (Fig. 2B). A secondary analysis of available anatomical data showed that bone-localized PMTs were primarily reported in the femur (42.0%, *n* = 89, *n* = 212), tibia (12.3%, *n* = 26, *n* = 212), and pelvic ring bones (21.7%, *n* = 46, *n* = 212), Supplementary Table 2A. Soft tissue tumors most frequently occurred in the thigh (24.1%, *n* = 54, *n* = 224) and foot (21.0%, *n* = 47, *n* = 224), Supplementary Table 2B. A small subset of PMT lesions (*n* = 23) was identified in visceral organs, all of which represented secondary metastatic lesions, primarily located in the lungs (*n* = 16).

Histopathological analysis showed that most PMTs were benign (93.4%, *n* = 353, *n* = 378), while malignant tumors accounted for only 6.6% (*n* = 25, *n* = 378). The overall predominant histological subtype was the mixed connective tissue (MCT) type (84.9%, *n* = 169, *n* = 199), with rarer subtypes such as hemangiopericytoma-like, osteoblastoma-like, chondromyxoid-fibroma-like, and others comprising 15.0% (*n* = 30, *n* = 199, Supplementary Table 1B). When comparing PMTs in different anatomical regions, no significant difference was found in the risk of malignancy between bone-localized and soft tissue tumors (bone: 2.8%, *n* = 4, *n* = 143 vs soft tissue: 1.5%, *n* = 3, *n* = 198; *P* = 0.41). However, PMTs detected in visceral organs were exclusively metastatic lesions and demonstrated a significantly higher risk of malignancy (86.7%, *n* = 13, *n* = 15) compared to tumors located outside visceral organs (3.1%, *n* = 11, *n* = 358), *P* < 0.001. Detailed analyses to assess the tumors’ local aggressiveness, risk of malignant transformation, or localization-specific risk of malignancy were not feasible due to the lack of longitudinal and detailed clinical data, but should be addressed in future prospective studies.

### Diagnostic approaches and biomarker profiles

Our analysis revealed a median time to diagnosis of 48 months (range: 0–456, *n* = 274, Fig. 1C), with diagnostic delays being significantly longer in bone-localized PMTs compared to tumors located in soft tissue (median: 48 vs 36 months, *P* = 0.012, Fig. 1D).

By analyzing diverse imaging modalities, MRI emerged as the overall gold standard in anatomical imaging for visualizing PMTs, primarily due to its high sensitivity and broad availability. MRI without contrast enhancement demonstrated tumor visibility in 94.1% of PMT cases (*n* = 159, *n* = 169), which increased to 100% (*n* = 52, *n* = 52) with contrast enhancement. Despite CT scans showing a significantly lower detection rate overall, they were significantly more effective in detecting bone-localized PMTs (89.6%, *n* = 60, *n* = 67) compared to soft tissue tumors (70.6%, *n* = 24, *n* = 34; *P* = 0.016). When combined with contrast enhancement, the detection rate of CT scans improved to 92.9% (*n* = 13, *n* = 14). However, CT scans generally played a secondary role in PMT diagnostics and were primarily used for assessing bone-localized PMTs or for surgical planning. PMT lesions were visible on plain radiography (X-rays) in 50% of cases (*n* = 31, *n* = 62). Other conventional imaging methods, such as ultrasonography (*n* = 28), were used exclusively for further evaluation of already palpable or otherwise noticeable masses rather than for primary imaging.

As the previously discussed imaging modalities are primarily suited for detailed visualization of specific anatomical regions, functional imaging techniques (e.g., nuclear medicine) allow for whole-body scanning and may therefore be used initially or to detect occult lesions. Positron emission tomography (PET) using various radionuclides – such as 18-fluoro-deoxyglucose (FDG) and Gallium-68 DOTA-TATE (^68^Ga-DOTATATE) – as well as scintigraphy with radioisotopes such as Technetium-99m (Tc99m) or 111-indium-octreotide (111In-Octreotide) were reported frequently in PMT diagnostics. Among these, ^68^Ga-DOTATATE PET demonstrated the highest accuracy, with 100% tumor visibility when performed (*n* = 99, *n* = 99). In contrast, FDG-PET visualized 67.6% of soft tissue PMTs (*n* = 25, *n* = 37) but up to 85.7% of bone-localized PMTs (*n* = 42, *n* = 49). Tc99m scintigraphy had an overall tumor visibility rate of 78.2% (*n* = 43, *n* = 55), while the 111In-Octreotide scan detected 63.3% of PMTs (*n* = 38, *n* = 60), highlighting their limited efficiency in both soft tissue and bone-localized PMT diagnostics.

Data on FGF-23 quantification were available for 311 patients, with 96.5% exhibiting elevated serum FGF-23 levels (*n* = 300, *n* = 311), highlighting its role as the most sensitive biomarker for PMT. No significant difference was present between soft tissue and bone-localized PMTs (*P* = 0.07). In addition, decreased serum phosphate concentrations were observed in 98.7% of patients (*n* = 386, *n* = 391), alongside increased renal phosphate excretion in 94.2% of cases (*n* = 179, *n* = 190). Levels of 1α,25-dihydroxycholecalciferol (vitamin D) were reduced in 70.6% of PMT patients (*n* = 89, *n* = 126), emphasizing the significant impact of PMTs on bone mineral homeostasis.

Due to missing and inconclusive data, the absence of uniform guidelines, and heterogeneous diagnostic approaches between institutes, a structured analysis of the importance of diagnostic biopsy – whether through incisional biopsy or radiologically guided fine-needle biopsy – was not feasible. However, histopathological reports of biopsy or resection specimens revealed certain markers such as CD56, vimentin, SSTR2A (somatostatin receptor 2A), FGFR1 (fibroblast growth factor receptor 1), and ERG (erythroblast transformation-specific related gene), which were indicative but not necessarily unique to PMT.

### Treatment strategies and outcome analysis in the literature cohort

Overall analysis of treatment-specific data revealed that 94.6% of patients with PMT underwent surgical intervention (*n* = 419, *n* = 443). Drug-based therapy was administered in 67.3% of patients (*n* = 165, *n* = 245), almost exclusively as an adjunct to surgery or non-surgical interventional procedures, rather than as stand-alone treatment. However, treatment-specific data with follow-up, allowing for outcome analysis, were available in only 260 patients, with 93.5% (*n* = 243) undergoing surgery and 6.5% (*n* = 17) receiving non-surgical treatment.

Surgical procedures were largely individualized and patient-tailored, including curettage, marginal or compartment resections, limb amputations, and other techniques. Defect reconstruction or prophylactic stabilization was required in some cases (*n* = 35), depending on the anatomical location and resection margins. Complete remission, defined as full relief of clinical symptoms, was achieved in 70.8% (*n* = 172) of surgically treated patients, while 25.1% (*n* = 61) experienced partial symptom relief. In contrast, only 29.4% (*n* = 5) of non-operatively managed patients achieved full remission, while 35.3% (*n* = 6) reported partial symptom relief (*P* < 0.001). Importantly, complete remissions in non-surgically treated patients were achieved only through local interventional procedures (e.g., catheter-guided embolization, cryoablation, or radiofrequency ablation) and not by drug-based therapy alone (20, 21, 22, 23, 24, 25).

Of the 243 surgically treated patients, resection status data were available for 61 patients, with additional localization data available for 29 soft tissue and 27 bone-localized PMTs. Complete resection was achieved in 72.1% (*n* = 44) of these cases, whereas 27.9% (*n* = 17) had incomplete resection. Full remission was significantly more frequent in patients who underwent complete resection (55.7%, *n* = 34) compared to those with incomplete resection (6.6%, *n* = 4; *P* = 0.001). Partial remission was observed in 20.5% (*n* = 9) following complete resection, while incomplete resection predominantly resulted in partial symptom relief (70.6%, *n* = 12).

When complete resection was achieved, full remission rates were comparable between soft tissue PMTs (80%, *n* = 16, *n* = 20) and bone-localized PMTs (81%, *n* = 17, *n* = 21). However, only in soft tissue PMTs did some cases still achieve full remission after incomplete resection. In bone-localized PMTs, the correlation between complete resection and full symptom relief was statistically significant (*n* = 27, Cramér’s V = 0.724, *P* < 0.001). Notably, among malignant tumors, only one case achieved complete remission (4%, *n* = 19), whereas 44% (*n* = 11) and 28% (*n* = 7) of cases showed no remission or only partial remission, respectively. Unfortunately, detailed data on resection status of this subgroup were available for only three malignant PMT tumors, limiting subsequent correlations with the preferred surgical intervention.

Medical treatment primarily focused on supplementation of phosphate (38.2%), calcium (13.2%), calcitriol (22.7%), or vitamin D (16.8%). Few cases reported attempts to modulate calcium receptors using cinacalcet (calcimimetic) (26) or to regulate growth hormones via octreotide (synthetic somatostatin analog) (27). Some patients received anti-cancer therapies, including doxorubicin (inhibiting DNA/RNA synthesis) (28), burosumab (a monoclonal antibody targeting FGF-23) (29, 30), or infigratinib (an FGF receptor inhibitor) (31). However, these treatments demonstrated limited long-term efficacy and sparse evidence supporting their effectiveness.

### Clinical and diagnostic characteristics of a single-center case series

#### Patient presentation and diagnostic workup

Our cohort included two males and two females, with a median age at diagnosis of 61.5 years (range: 52–70 years). Typically, patients presented with a combination of generalized pain (100%), pathological fractures (75%), muscle weakness (50%), and movement restriction (50%). In two of three cases, pathological fractures occurred at sites distant from the tumor. In addition, two patients with PMTs in the foot reported swelling (50%) at the tumor site. All four tumors were located in the lower limb, with one confined to the greater trochanter of the femur, one involving both bone and soft tissue of the proximal femur diaphysis, and two soft-tissue PMTs of the foot. The median tumor volume was 6.3 cm^3^ (range: 4.9–84.2 cm^3^) (Tables 1 and 2).

**Table 1:** Surgical management and clinical overview of four representative patients with PMT. All patients underwent surgical resection as primary treatment. Bone and/or soft tissue infiltration was observed, with resection margins ranging from R0 to R1. No metastases were reported. Three patients achieved no evidence of disease (NED), while one patient remained alive with disease (AWD) due to R1 status. Remission status was complete in soft tissue cases and partial in bone-infiltrating tumors.

Patient	Tumor location	Tumor infiltration	Primary treatment	Type of treatment	Resection status (margin)	Local recurrence	Metastasis	Remission	Final status
1	Femur (greater trochanter)	Bone	Surgery	Resection + osteosynthesis	R0 (0.1–0.3 cm)	No	No	Partial	NED
2	Femur (proximal)	Bone & soft tissue	Surgery	Resection + osteosynthesis	R0 (0.5–0.9 cm)	No	No	Partial	NED
3	Foot	Soft tissue	Surgery	Resection	R0 (0.1–0.4 cm)	No	No	Complete	NED
4	Foot	Soft tissue	Surgery	Resection	R1 (<0.1 cm)	-	No	Complete	AWD

**Table 2:** Histopathological and molecular characteristics of four representative patients with PMT. All tumors were histologically benign, with variable sizes and anatomical locations. Immunohistochemical profiles showed heterogeneous expression of markers such as CD56, ERG, and somatostatin receptor. FN1-FGFR1 RNA fusion was detected in one patient, while another tested negative.

Patient	Tumor location	Tumor size (cm^3^)	Dignity	Positive (+) immunohistochemical expression	Negative (−) immunohistochemical expression	FN1-FGFR1 RNA-fusion
1	Femur (bone)	5.2	Benign	CD56, ERG, MIB-1 (max. 8%)	Somatostatin-receptor, CD31, CD34	-
2	Femur (bone and soft tissue)	7.4	Benign	CD56, ERG, SATB2, somatostatin-receptor, INI1	CD14, CD31, CD34, CD68, H3F3A	Negative
3	Foot (soft tissue)	84.2	Benign	-	-	-
4	Foot (soft tissue)	4.9	Benign	CD56, ERG, SSTR2A	-	Positive

**Figure 3:**
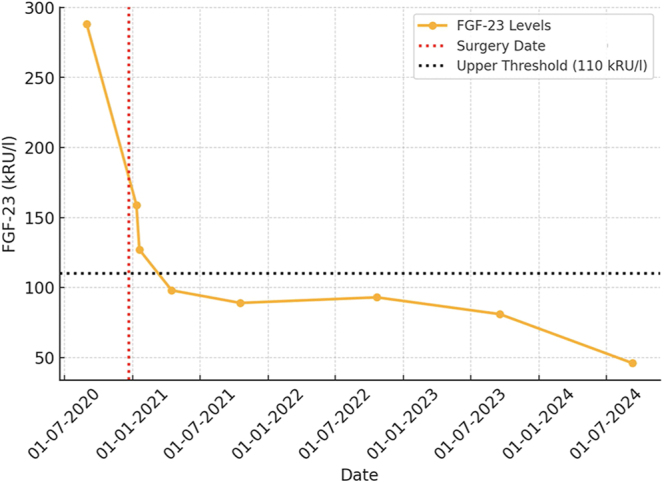
Longitudinal FGF-23 levels before and after PMT tumor resection. FGF-23 serum levels (orange line) markedly declined following surgical tumor resection (red dashed line) and remained below the upper reference threshold of 110 kRU/L (black dashed line) over the long-term follow-up.

The median time from symptom onset to diagnosis was 3.8 years (45.5 months; range: 1–60 months). At diagnosis, hypophosphatemia with normocalcemia was the typical presentation, with three out of four patients exhibiting reduced phosphorus (median: 0.3 mmol/L; range: 0.3–0.7 mmol/L) and normal calcium levels (median: 2.3 mmol/L; range: 2.29–2.31 mmol/L) (Table 3). Preoperative FGF-23 levels were elevated in all cases (median: 427.5 kRU/L; range: 130–514 kRU/L) and reduced to regular values at the latest follow-up (median: 62.5 kRU/L; range: 46–78 kRU/L) (Table 3, Fig. 3).

**Table 3:** Laboratory values in four patients with PMT before and after tumor resection. FGF-23 levels decreased postoperatively in all patients with available follow-up data. Serum phosphorus levels normalized in patients 1–3 after surgery. Calcium levels remained stable or increased slightly within the normal range across all measured time points. Last FU refers to the last available follow-up measurement.

Patient	FGF-23			Phosphorus			Calcium		
	Pre-OP	Post-OP	Last FU	Pre-OP	Post-OP	Last FU	Pre-OP	Post-OP	Last FU
1	288	159	46	0.3	0.87	0.82	2.29	2.31	2.43
2	567	79	79	0.4	0.8	1.2	2.31	2.46	2.54
3	514	-	-	0.7	0.91	0.78	2.33	2.45	2.58
4	130	78	78	-	-	-	2.3	-	-

**Figure 4:**
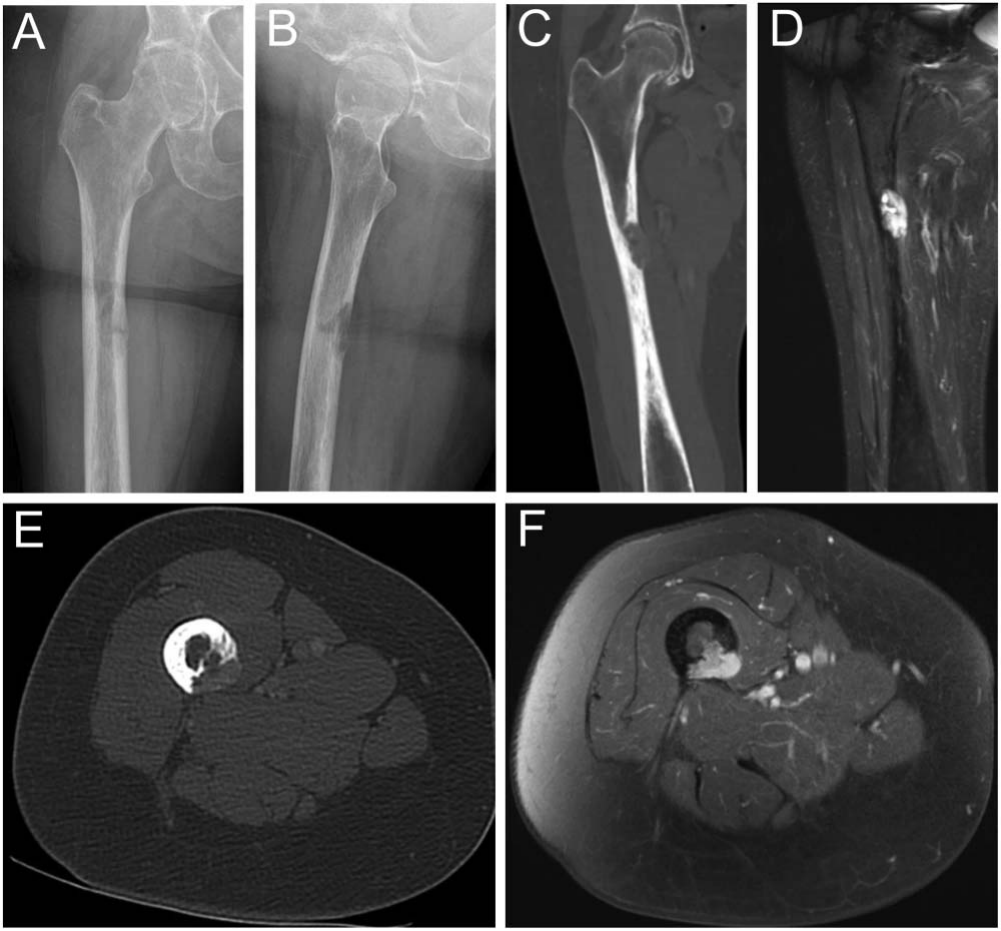
Radiological images using X-ray, CT, and MRI. (A and B) X-rays showing an osteolytic lesion with cortical erosion, accompanied by a pathological fracture. (C and E) CT scans demonstrating the tumor lesion with cortical bone destruction. MRI scans showing characteristic T2 hyperintensity and contrast enhancement (D), along with extraosseous soft tissue tumor formation (F).

**Figure 5:**
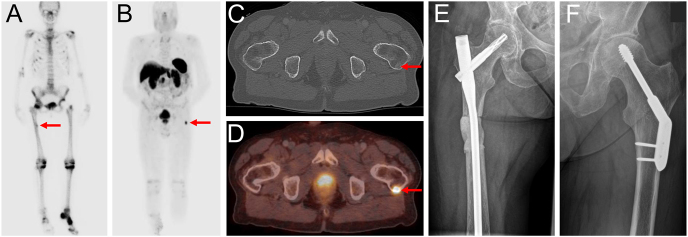
Scintigraphy and PET/CT, as well as postoperative radiographs following osteosynthetic stabilization. (A) Tc-99m nuclear scintigraphy demonstrating significant radiotracer uptake at the primary tumor site, along with multiple additional sites due to pathological fractures and impaired bone mineralization. (B, C, D) Preoperative imaging with ^68^Ga-DOTATATE PET/CT showing intense and specific uptake at the tumor site. (E and F) Postoperative radiographs showing osteosynthetic stabilization using a femoral nail and a dynamic hip screw following tumor resection. A cement spacer was used for defect filling (E). Red arrows indicating tumor lesion.

**Figure 6:**
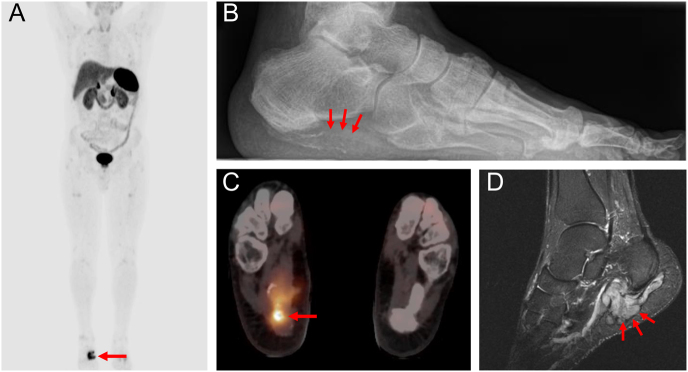
Multimodal imaging approach for localization of a PMT in the foot. Initial plain radiograph (B) revealed a subtle, partially calcified lesion within the soft tissue close to the calcaneus (red arrows). Functional imaging using ^68^Ga-DOTATATE PET/CT demonstrated intense radiotracer uptake at the lesion site (C), and whole-body PET localized the tumor to the right foot with high sensitivity (A). Anatomical imaging with MRI (D) showed a T2w hyperintense, contrast-enhancing, well-demarcated lesion consistent with a PMT.

Three patients underwent MRI before definitive diagnosis, with all lesions showing T2 hyperintensity and contrast enhancement as common and characteristic features. Lesions appeared partially calcified with cortical destruction of the bone and soft tissue infiltration in some cases (Table 4). In addition, one patient had an X-ray and CT scan where the tumor lesion appeared lytic along with cortical erosion, a pathological fracture, and extra-osseous soft tissue infiltration (Fig. 4). Nuclear Tc-99m scintigraphy was performed in three patients, showing significant nuclide uptake of the tumor lesions and multiple other locations due to pathological fractures and impaired bone mineralization. In two patients, 68-Ga-DOTATATE-PET/CT was performed before biopsy, after years of nonspecific symptoms and repeated interdisciplinary presentations, and demonstrated avid uptake of the tumor lesions (Table 4, Fig. 5). In one patient, PET/CT was performed only postoperatively, as PMT was initially not suspected and biopsy was undertaken directly. Given the mostly small tumor size and variable localization, the complementary value of combining plain radiography, functional imaging, and subsequent anatomical imaging should be emphasized in the diagnostic workup of PMT (Fig. 6).

**Table 4:** Radiological findings in four representative patients with PMT. MRI consistently revealed T2-weighted hyperintensity and contrast enhancement in patients with soft tissue involvement. CT and X-ray identified osteolytic changes and cortical erosion in cases with bone infiltration. Functional imaging with ^68^Ga-DOTATATE PET/CT and Tc-99m scintigraphy showed radiotracer uptake at the tumor site in all assessable patients, supporting the diagnosis and localization of phosphaturic mesenchymal tumors.

Patient	Tumor location	MRI	CT	X-ray	68Ga- DOTATATE-PET/CT	Tc-99m-SCINTIGRAPHY
1	Femur (bone)	-	-	-	Nuclide uptake at tumor lesion	Nuclide uptake at tumor lesion
2	Femur (bone and soft tissue)	T2w hyperintensity	Osteolysis	Osteolysis	-	Nuclide uptake at tumor lesion
		Contrast enhancement	Cortical erosion	Cortical erosion		
			Pathological fracture			
3	Foot (soft tissue)	T2w hyperintensity	-	-	Nuclide uptake at tumor lesion	Nuclide uptake at tumor lesion
		Contrast enhancement				
		Polylobulated, partially calcified mass				
4	Foot (soft tissue)	T2w hyperintensity	-	-	-	-
		Contrast enhancement				

Overall, diagnosis in all cases was based on a combination of clinical symptoms, laboratory findings, and at least one imaging modality. All four patients subsequently underwent diagnostic biopsy for definitive histopathological verification before surgical treatment. In addition, an interdisciplinary workflow, including discussion within our comprehensive cancer center tumor board, was required in all cases to ensure accurate diagnosis and appropriate treatment planning.

#### Histopathological and molecular findings

**Figure 7:**
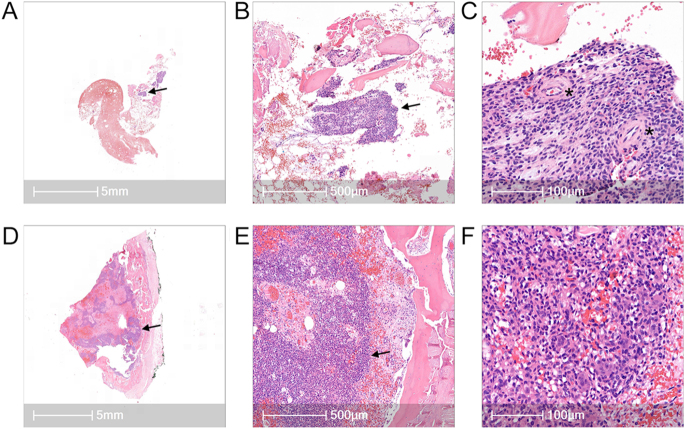
Histopathological images of PMT. (A, B, C) Biopsy specimen shows small tumor area (arrow) composed of spindle-shaped mesenchymal cells next to partly vascular (*) and chondroid proliferations in the fatty tissue along with bone particles. No evidence of malignant potential of the tumor. (D, E, F) Resection specimen shows an intraosseous, richly vascularized (focally hemangiopericytoma-like) tumor area (arrow) composed of typical spindle-shaped mesenchymal cells. Partly sclerosed sections and intermixed multinucleated giant cells consistent with a PMT. No evidence for histological criteria of malignancy.

PMT samples exhibited distinct and characteristic features on histopathological examination. Microscopically, the tumor areas were primarily composed of spindle-shaped mesenchymal cells, typical of PMTs. No significant cytologic atypia, mitotic activity, or other malignant features were observed in any sample (Fig. 7, Table 2). Immunohistochemically, tumor cells showed nuclear ERG and CD56 positivity in three out of four patients, consistent with PMTs (Table 2). RNA panel sequencing (TruSight RNA Fusion Panel, Illumina Inc., USA) was performed in two cases, detecting an FGFR1 RNA fusion in one sample. In line with the existing literature, two PMTs also expressed additional biomarkers, including SATB2 (special AT-rich sequence-binding protein 2) and SSTR2A. PMT tumor cells generally lacked immunoreactivity for endothelial markers such as CD34 and CD31 when these markers were included in immunohistochemical examinations (Table 2).

#### Surgical management and clinical outcomes

Surgery, as the first-line treatment, was performed in all patients, with two patients additionally receiving phosphorus and vitamin D supplementation. Depending on the anatomical localization, an interdisciplinary surgical approach involving orthopedic and plastic tumor surgeons was required, particularly for PMTs infiltrating bone and surrounding soft tissue. All patients underwent marginal resection of the tumor mass, achieving tumor-free margins (R0) of 0.1–0.9 cm in three patients (Table 1). In one patient, histopathological examination revealed an R1 resection status, indicating small tumor residues within margins of <0.1 cm. In two patients with PMTs located in the proximal femur, additional osteosynthetic stabilization was performed (Fig. 5). During follow-up, no local recurrence was observed in any patient (Table 1). However, in one patient with histological R1 resection status, a follow-up MRI after 1 year showed no clear signs of recurrence but could not definitively exclude minor residual tumor persistence. Distant metastases were not detected in any of the patients. At the last follow-up, three patients showed no evidence of disease, and one patient was alive with disease (R1 resection status). Two patients reported complete remission of symptoms at the follow-up visits to our outpatient clinic, while two others experienced at least partial symptom relief (Table 1). These patients, suffering from bone-localized PMTs, underwent osteosynthesis for prophylactic stabilization and showed minor movement limitations, likely attributable to postoperative scarring in the region of the surgical approach.

## Discussion

PMTs represent a rare entity frequently associated with TIO, a condition driven by excess FGF-23 production (32). Despite recent advances in diagnostic imaging and an increasing number of reports on PMT-specific biomarkers or molecular features, achieving a time-efficient diagnosis remains challenging.

Our findings reaffirm the non-specific and often misleading clinical presentation of PMTs, with bone pain, pathological fractures, and functional impairment being the predominant symptoms (2, 11). Although common, these features do not indicate the tumor location and frequently contribute to diagnostic delays. In our cohort, the median time to diagnosis exceeded 3 years, consistent with the previous literature reporting comparable prolonged diagnostic latency (12, 16, 32). Notably, our analysis showed that bone-localized PMTs were associated with a significantly longer delay than soft tissue tumors, possibly due to their less accessible and noticeable anatomical locations. Consistent with earlier studies, PMTs were nearly equally distributed between bone and soft tissue, with a preference for lower extremities (33, 34). Visceral organ involvement, with the lung as the most common site, was rare and exclusively metastatic, in line with existing reports indicating the absence of primary PMTs in these organs (32, 33). Histologically, most PMTs were benign, with the MCT variant being predominant. Malignancy was exceedingly rare, occurring in 6.6% of all PMTs, which is comparable to rates reported in previous studies (33, 35, 36). Metastases of PMTs almost invariably demonstrate malignant histological features (1, 37, 38); however, two cases of histologically ‘benign’ metastases have been reported in the literature (34, 39). Importantly, despite lacking classical criteria of malignancy, these tumors ultimately exhibited malignant potential and should, therefore, be interpreted and treated accordingly. Given the absence of longitudinal and detailed clinical data of the presented study, analyses of local aggressiveness, risk of malignant transformation, and localization-specific risk of malignancy were not feasible and should be the focus of future prospective studies.

Radiological imaging remains central to PMT diagnosis, with plain radiography (X-rays) often performed as the first radiological technique in symptomatic patients due to its fast and low-budget accessibility. Common features such as generalized osteopenia, pathological fractures, calcifications, or cortical erosions may be visible but are not necessarily specific to PMT (40). Since most tumors are generally small, radiographs as a single modality may not be sufficient for tumor localization. Hence, further anatomical and functional imaging techniques are usually required (41). CT scans present as a useful modality primarily in bone-associated tumor lesions and for precise surgical planning to achieve tumor-free resection margins. Our findings also confirm that MRI, particularly when contrast enhancement was applied, was demonstrated as one of the most sensitive modalities for PMT localization, as reported by others (13, 14). Due to various alternative diagnoses that may present with similar radiological appearance, a multi-modality approach to imaging diagnosis is mandatory (40). Thus, functional (nuclear medicine) imaging techniques may be beneficial in localizing even very small and occult PMTs by whole-body scanning, followed by detailed anatomical imaging (e.g., MRI). Out of these modalities, 18F-FDG-PET, Tc-99m scintigraphy, and 111In-Octreotide scans showed variable sensitivity (63.3–85.7%) in our analysis, supporting the preferential use of somatostatin receptor-based imaging for suspected PMTs (42, 43). Both our review and case series demonstrated the superior efficiency of ^68^Ga-DOTATATE PET/CT with >90% sensitivity in the localization of PMT, in line with previous reports (44, 45). The choice of molecular imaging highly depends on the local availability of the institution, emphasizing the urgency of referring patients to comprehensive cancer centers if PMT is suspected. However, despite characteristic findings on functional imaging, histopathological confirmation by biopsy remains essential for definitive diagnosis of PMT and should always be sought.

Since PMTs frequently cause serum alterations due to FGF-23 secretion, conventional blood diagnostics are crucial, particularly in PMT detection and long-term surveillance. Nearly all analyzed PMT cases demonstrated elevated serum FGF-23 levels and hypophosphatemia, reinforcing the diagnostic relevance of FGF-23 elevation in raising suspicion for a possible PMT, as previously described (12, 32, 46). The consistency of FGF-23 levels declining after complete tumor resection, as observed in both our systematic review and case series, highlights its additional role as a reliable biomarker for disease activity and treatment response (9, 34). Histopathological analyses, including immunohistochemistry and RNA fusion testing of the presented case series, revealed expression of CD56, ERG, and SSTR2A, along with molecular-genetic alterations such as FGFR1 RNA fusions. These results align with our systematic review and previously described molecular features of PMTs (6, 47, 48, 49). However, the immunohistochemical and genetic alterations are not present in all PMTs; they lack specificity and should, therefore, always be interpreted in conjunction with clinical and radiological findings.

Based on our institutional data and experience, we recommend performing a diagnostic biopsy using minimally invasive, image-guided procedures when a PMT is suspected and the tumor location has been verified. This approach allows for a reliable histopathological diagnosis before definitive surgical treatment while minimizing procedural morbidity. Given the rarity of PMTs, histopathological examination should be performed by board-certified pathologists within a comprehensive cancer center to ensure diagnostic accuracy.

Surgical resection remains the cornerstone of definitive PMT treatment, with our review showing significantly higher remission rates in patients undergoing complete resection (R0) compared to those with incomplete or no resection. This confirms earlier findings emphasizing the importance of achieving clear margins (35, 36). The outcomes of our institutional case series reinforce these findings: three of four patients achieved R0 resections and remained disease-free during follow-up. Only one patient, who had an R1 resection, was alive with residual disease but remained clinically stable. These results underscore the importance of achieving tumor-free margins during surgery to optimize long-term outcomes. Notably, by comparing different anatomical tumor sites, our study supports previous reports (36) and highlights a stronger correlation between complete resection and remission for bone-localized compared to soft tissue PMTs, underlining that anatomical sites influence surgical outcomes. Even though complete surgical tumor resection seems to be favorable, one should always consider anatomical and functional factors. As shown in our case series, bone-localized PMTs with complete surgical resection may need osteosynthetic stabilization, which could be associated with greater morbidity and loss of some limb functions. On a final note, malignant PMT tumors showed a full remission rate of only 4% (*n* = 1, *n* = 19) and seem to be associated with an even worse outcome. Given the limited number of malignant cases and the lack of comprehensive clinical information in the present study, definitive conclusions regarding the potentially more difficult treatment of malignant tumors could not be drawn. Future studies with larger malignant cohorts or detailed longitudinal clinical and pathological data are warranted to clarify the clinical and biological differences between benign and malignant PMT tumors.

Non-surgical treatments, such as phosphate or vitamin D supplementation (50, 51), radiotherapy (52, 53), or ablation therapies (54), demonstrated limited and inconsistent effectiveness (55). While agents such as Burosumab (anti-FGF23 antibody) show promise in targeting FGF-23 directly, evidence from long-term follow-up remains sparse (56). Thus, such options should be considered primarily in cases where surgery is not feasible, refused by the patient, or in metastatic disease (21).

### Limitations

The systematic review is limited due to the retrospective nature of many of the included studies and the heterogeneity in reported data. Many of the included studies did not provide long-term follow-up or detailed outcome documentation, which may affect the accuracy of sub-analyses. In addition, the general rarity of PMTs and available publications, mostly as case reports, limit the level of evidence.

Our case series has a very small sample size of only four patients, limiting the generalizability of the presented findings and precluding meaningful statistical analysis. Variable follow-up durations and incomplete data may obscure long-term outcomes such as recurrence or delayed remission. Further, the limited number of cases holds the risk of missing clinically relevant events, such as malignant transformation or late disease progression.

Despite these limitations, our study provides valuable insights into the clinical management of PMT. Given the rarity of these tumors and the diagnostic delays frequently encountered, studies like this are essential to raising awareness, guiding clinical decision-making, and improving patient outcomes.

## Conclusion

- Despite improvements in imaging and biomarker availability, delayed diagnoses remain a relevant issue, particularly for bone-localized PMTs.
- Clinicians should be alert for patients with unexplained osteomalacia, particularly if accompanied by hypophosphatemia and elevated FGF-23 levels.
- If PMT is suspected, focused imaging should be performed early in the diagnostic process, for example, using a whole-body ^68^Ga-DOTATATE PET/CT scan, to identify otherwise occult lesions.
- Surgical resection with negative margins remains the most effective curative approach, with markedly better outcomes than non-operative therapies.

## Supplementary materials





## ICMJE Statement of Interest

The authors declare that there is no conflict of interest that could be perceived as prejudicing the impartiality of the work reported.

## Funding Statement

This research did not receive any specific grant from funding agencies in the public, commercial, or not-for-profit sectors.

## Data availability

The datasets generated and/or analyzed during this study are not publicly available due to patient confidentiality and institutional restrictions, but are available from the corresponding author on reasonable request.

## Ethics approval and informed consent statement

The local ethics committee of the University of Freiburg approved the data evaluation (protocol 23-1526-S1-retro). Written informed consent was obtained from all patients treated at the University Medical Center Freiburg, Germany.
