# Supplementary material for: Surgical treatment as a key determinant of outcome in phosphaturic mesenchymal tumors of the bone and soft tissue: a systematic review and case series
Source: EFORT Open Rev. 2025 Nov 3;10(11):829–41. doi: 10.1530/EOR-2025-0100 (PMC12587032; doi:10.1530/EOR-2025-0100)
Supplement: Supplementary file 1 [file supplementary_table_1.pdf]

A

Bone location	Number (N)	Frequency (%)
Femur	89	41,98%
Tibia	26	12,26%
Humerus	16	7,55%
Ischium	14	6,60%
Ilium	14	6,60%
Acetabulum	9	4,25%
Lumbal vertebrae	8	3,77%
Fibula	8	3,77%
Sacrum	7	3,30%
Radius	5	2,36%
Rib	4	1,89%
Scapula	3	1,42%
Thoracic vertebrae	3	1,42%
Metatarsal bones	2	0,94%
Ulna	2	0,94%
Pubis	2	0,94%

B

Soft tissue location	Number (N)	Frequency (%)
Thigh	54	24,11%
Foot	47	20,98%
Leg	10	4,46%
Popliteal fossa	9	4,02%
Knee	9	4,02%
Chest wall	9	4,02%
Buttock	8	3,57%
Inguinal region	8	3,57%
Groin	7	3,13%
Lower leg	6	2,68%
Gluteal region	6	2,68%
Ankle	5	2,23%
Shoulder	5	2,23%
Forearm	4	1,79%
Back	4	1,79%
Finger	4	1,79%
Pleura	3	1,34%
Iliopsoas muscle	3	1,34%
Hip region	3	1,34%
Palm	3	1,34%
Gastrocnemius	3	1,34%
Trapezius	2	0,89%
Perineal region	2	0,89%
Quadriceps	2	0,89%
Upper arm	2	0,89%
Thenar	2	0,89%
Abdominal wall	2	0,89%
Mediastinum	2	0,89%
