# Supplementary material for: Surgical treatment as a key determinant of outcome in phosphaturic mesenchymal tumors of the bone and soft tissue: a systematic review and case series
Source: EFORT Open Rev. 2025 Nov 3;10(11):829–41. doi: 10.1530/EOR-2025-0100 (PMC12587032; doi:10.1530/EOR-2025-0100)
Supplement: Supplementary file 2 [file supplementary_table_2.pdf]

A

Rare Symptoms	Number (N)
Fatigue	31
Osteopenia	11
Muscle spasm	3
Loss of Weight	3
Paresthesia	2
Tenderness	2
Cramps	2
Paresis	2
Spasms	2

B

Rare histological subtypes*	Number (N)
Hemangiopericytoma-like	14
Osteoblastoma-like	5
Chondromyxoidfibroma-like	3
Non-ossifying fibroma-like	2
Neurofibroma-like	1
Neuroma-like	1
Spindle cell mesenchymal-like	1
Giant cell granuloma-like	1
Spindle sarcomatous-like	1
